# Clinical course and outcome of patients with acute pulmonary embolism rescued by veno-arterial extracorporeal membrane oxygenation: a retrospective review of 21 cases

**DOI:** 10.1186/s13019-020-01347-0

**Published:** 2020-10-02

**Authors:** Yen-Yu Chen, Yin-Chia Chen, Chia-Chen Wu, Hsu-Ting Yen, Kwan-Ru Huang, Jiunn-Jye Sheu, Fan-Yen Lee

**Affiliations:** Division of Thoracic and Cardiovascular Surgery, Department of Surgery, Kaohsiung Chang Gung Memorial Hospital and Chang Gung University College of Medicine, 123, Ta-Pei Road, NiaoSung, Kaohsiung City, 83301 Taiwan

**Keywords:** Acute pulmonary embolism, Extracorporeal membrane oxygenation, Cardiopulmonary resuscitation, Cardiac arrest

## Abstract

**Background:**

Veno-arterial extracorporeal membrane oxygenation (ECMO) is increasingly being utilized in patients with massive pulmonary embolism (PE). However, the efficacy and the safety remain uncertain. This study aimed to investigate clinical courses and outcomes in ECMO-treated patients with acute PE.

**Methods:**

Twenty-one patients with acute PE rescued by ECMO from January 2012 to December 2019 were retrospectively analysed. Clinical features, laboratory biomarkers, and imaging findings of these patients were reviewed, and the relationship with immediate outcome and clinical course was investigated.

**Results:**

Sixteen patients (76.2%) experienced refractory circulatory collapse requiring cardiopulmonary resuscitation (CPR) or ECMO support within 2 h after the onset of cardiogenic shock, and none could receive definitive reperfusion therapy before ECMO initiation. Before or during ECMO support, more than 90% of patients had imaging signs of right ventricular (RV) dysfunction. In normotension patients, the computed tomography (CT) value was a valuable predictor of rapid disease progression compared with cardiac troponin I level. Ultimately, in-hospital death occurred in ten patients (47.6%) and 90% of them died of prolonged CPR-related brain death. Cardiac arrest was a significant predictor of poor prognosis (*p* = 0.001).

**Conclusions:**

ECMO appears to be a safe and effective circulatory support in patients with massive PE. Close monitoring in intensive care unit is recommended in patients with RV dysfunction and aggressive use of ECMO may reduce the risk of sudden cardiac arrest and improve clinical outcome.

## Background

Acute pulmonary embolism (PE) is a major cause of sudden death worldwide and is often difficult to diagnose because of the variable clinical presentations, particularly in patients with pre-existing cardiopulmonary disease [1]. Massive or high risk PE, defined as PE resulting in hemodynamic instability, carries a high mortality rate, ranging from 30% for patients with cardiogenic shock to 70% for those that required cardiopulmonary resuscitation (CPR) [2]. For patients with massive PE, primary reperfusion therapy, including thrombolysis, surgical embolectomy, and catheter-directed therapies is recommended as first-line therapy by international guidelines [3, 4]. However, in clinical practice, many patients cannot receive reperfusion therapy because of major clinical instability or a concern with an increased risk of bleeding, especially in patients with recent major surgery or trauma [5].

For critically ill patients, veno-arterial extracorporeal membrane oxygenation (ECMO) is a reliable mechanical circulatory support device to decrease right ventricular (RV) volume overload, stabilize hemodynamic status, and provide gas exchange [6], and may be considered as either a bridge to definitive reperfusion therapy or as a stand-alone treatment strategy [7]. However, evidence for use of ECMO is limited to small case series [6–8] and the role of ECMO is not established.

Herein, we describe our experience with ECMO in patients with massive PE, and report their clinical courses and short-term outcomes. Moreover, we aim to identify the predictor associated with poor prognosis of these patients.

## Methods

### Patient selection and data collection

We retrospectively analysed our institution’s ECMO database to identify patients requiring circulatory support with ECMO for suspected or confirmed PE between January 2012 and December 2019. According to the American Heart Association and European Society of Cardiology guidelines, PE was diagnosed using the diagnostic strategy tools [3, 4]. Patients presenting with hemodynamic instability were defined as massive PE, and imaging and laboratory parameters were used to distinguish submassive and low-risk PE [3]. In total, 421 consecutive patients were treated for acute PE during the 8-year study period. Seventy-one (16.9%) patients had massive PE with a mortality rate of 71.8%, of whom 21 requiring circulatory support with ECMO were included in this study. Baseline characteristics and clinical variables were collected from patients’ medical records, including age, sex, body mass index, smoking history, comorbidities, predisposing factors for venous thromboembolism, and presenting symptoms and signs. The time of presence of initial symptoms, refractory circulatory collapse (cardiogenic shock or cardiac arrest), and ECMO initiation was recorded. The prognostic values for PE were collected, including arterial blood gas data, cardiac troponin I [9], electrocardiogram (ECG), echocardiography, and computed tomography (CT). Cardiac troponin I level ≥ 0.04 ng/mL (10% the coefficient of variation levels) was defined as elevated cardiac troponin I level in our institution. ECG findings of RV strain [10, 11] were defined as by the presence of at least one of the following: 1) S1Q3T3 pattern, 2) complete or incomplete RBBB, 3) inverted T waves in leads V1–V4, 4) ST elevation in lead aVR, 5) qR pattern in lead V1. Echocardiographic findings of RV dysfunction [12, 13] before or after ECMO initiation were defined as by the presence of at least one of the following: 1) RV dilatation or dysfunction, 2) tricuspid annular plane systolic excursion < 16 mm, 3) D-shaped left ventricle, 3) dilated inferior vena cava without respiratory variation. At CT scan, before or after ECMO initiation, RV dysfunction [14] was defined as a right-to-left ventricular (RV/LV) diameter ratio ≥ 0.9 measured in the transverse or four-chamber view.

### Indication, technique, and weaning of ECMO

All patients with persistent profound hypotension or cardiac arrest were placed on ECMO by cardiovascular surgeons. Cannulation was performed under ultrasound guidance or surgical exploration, with a 21-French to 23-French venous cannula inserted in the femoral vein, and a 15-French to 16.5-French arterial cannula inserted in the femoral artery. A 6-French distal perfusion catheter was placed in the ipsilateral superficial femoral artery for patients with significant leg ischemia. Anticoagulant therapy with unfractionated heparin was mandatory unless contraindicated, with a target activated clotting time of 180 to 220 s. Intravenous antihypertensive agents were administered to maintain systolic blood pressure below 120 mmHg to prevent brain hemorrhage, and inotropes or vasopressors were used to achieve target mean arterial pressure of 60 to 80 mmHg. Once the patient had markedly recovered after definitive treatment, such as anticoagulation therapy, or thrombolysis, ECMO was weaned by gradually reducing circuit flow till 1 l per minute. The cannulas were removed if the patient could maintain hemodynamic stability and acceptable pulmonary artery pressure while the flow was temporarily interrupted. If the patient had a brain death, ECMO support was withdrawn early.

### Outcomes variables

The primary outcome was in-hospital mortality. Secondary outcomes included acute neurologic complications (profound comatose states, seizures, brain hemorrhage, brain infarction, and brain death), acute kidney injury that required renal replacement therapy, major bleeding that required endoscopic, endovascular or surgical intervention, and ECMO-related complications, including vascular access site complications and leg ischemia.

### Statistical analysis

Continuous variables were expressed as mean ± standard deviation and were analysed using Student’s T test or Mann-whitney U-test. Categorical variables were expressed as number (percentage) and were compared using the Chi-squared test. We used RV/LV diameter ratio measurement on CT and cardiac troponin I prior to refractory circulatory collapse for predicting the clinical course. Correlations between the CT values or the cardiac biomarker and the time interval from diagnosis to persistent profound hypotension or cardiac arrest were evaluated using Pearson’s correlation or Spearman correlation. *P* values less than 0.05 were considered to indicate significant differences. The receiver operating characteristic curve analysis and area under the curve were used and the highest Youden’s index was used to determine the cut-off values for predicting in-hospital mortality. All analyses were conducted using SPSS software (version 19.0; SPSS Inc., Chicago, IL).

## Results

### Patient demographic characteristics and clinical presentations

Twenty-one patients were identified with a mean age of 54.8 years (range, 26 to 83 years). Nineteen patients had visualized pulmonary artery thrombus by using CT scan, one patient had a diagnosis based on the presence of impaired gas exchange, hemodynamic instability, and right heart thrombus, and one patient was diagnosed based on high clinical probability and echocardiographic finding of RV dilation without other plausible cause. The baseline clinical and demographic characteristics of these patients were summarized in Table 1. All except one patient had provoking factors for PE, included major surgery (61.9%), active cancer (33.3%), and prolonged bed rest (23.8%). More than half of the patients experienced worsening dyspnea (61.9%) and unexplained tachycardia (52.4%) before diagnosis of PE. Two hospitalized patients remained asymptomatic until sudden cardiac arrest occurred and four patients presented with cardiogenic shock on arrival to the emergency department.

**Table 1:** Baseline clinical and demographic characteristics (*N* = 21)

Characteristic	Value
Age, years	54.8 ± 16.1
Male sex, n (%)	15 (71.4)
Obesity (BMI > 27 kg/m^2^)	6 (28.6)
Hypertension, n (%)	7 (33.3)
Diabetes mellitus, n (%)	2 (9.5)
Smoking, n (%)	6 (28.6)
Hyperlipidemia, n (%)	2 (9.5)
Congestive heart failure, n (%)	1 (4.8)
Chronic kidney disease, n (%)	1 (4.8)
History of stroke, n (%)	1 (4.8)
Liver cirrhosis, n (%)	0 (0.0)
Predisposing factors for PE	
Clinical sign of DVT, n (%)	3 (14.3)
Major surgery, n (%)	13 (61.9)
Major trauma, n (%)	3 (14.3)
Active cancer, n (%)	7 (33.3)
Recent myocardial infarction, n (%)	1 (4.8)
History of VTE, n (%)	2 (9.5)
ICU length of stay > 72 h, n (%)	5 (23.8)
Immobilization of the lower limbs, n (%)	3 (14.3)
Postpartum period, n (%)	2 (9.5)
Clinical presentation	
Tachycardia, n (%)	11 (52.4)
Fever, n (%)	6 (28.6)
Chest pain, n (%)	2 (9.5)
Cold sweating, n (%)	7 (33.3)
Dyspnea, n (%)	13 (61.9)
Hemoptysis, n (%)	2 (9.5)
Syncope, n (%)	1 (4.8)
Altered mental status, n (%)	1 (4.8)
Hypotension or cardiac arrest, n (%)	6 (28.6)

*BMI* body mass index, *DVT* deep vein thrombosis, *ECMO* veno-arterial extracorporeal membrane oxygenation, *PE* pulmonary embolism, *ICU* intensive care unit, *VTE* venous thromboembolism

**Fig. 1:**
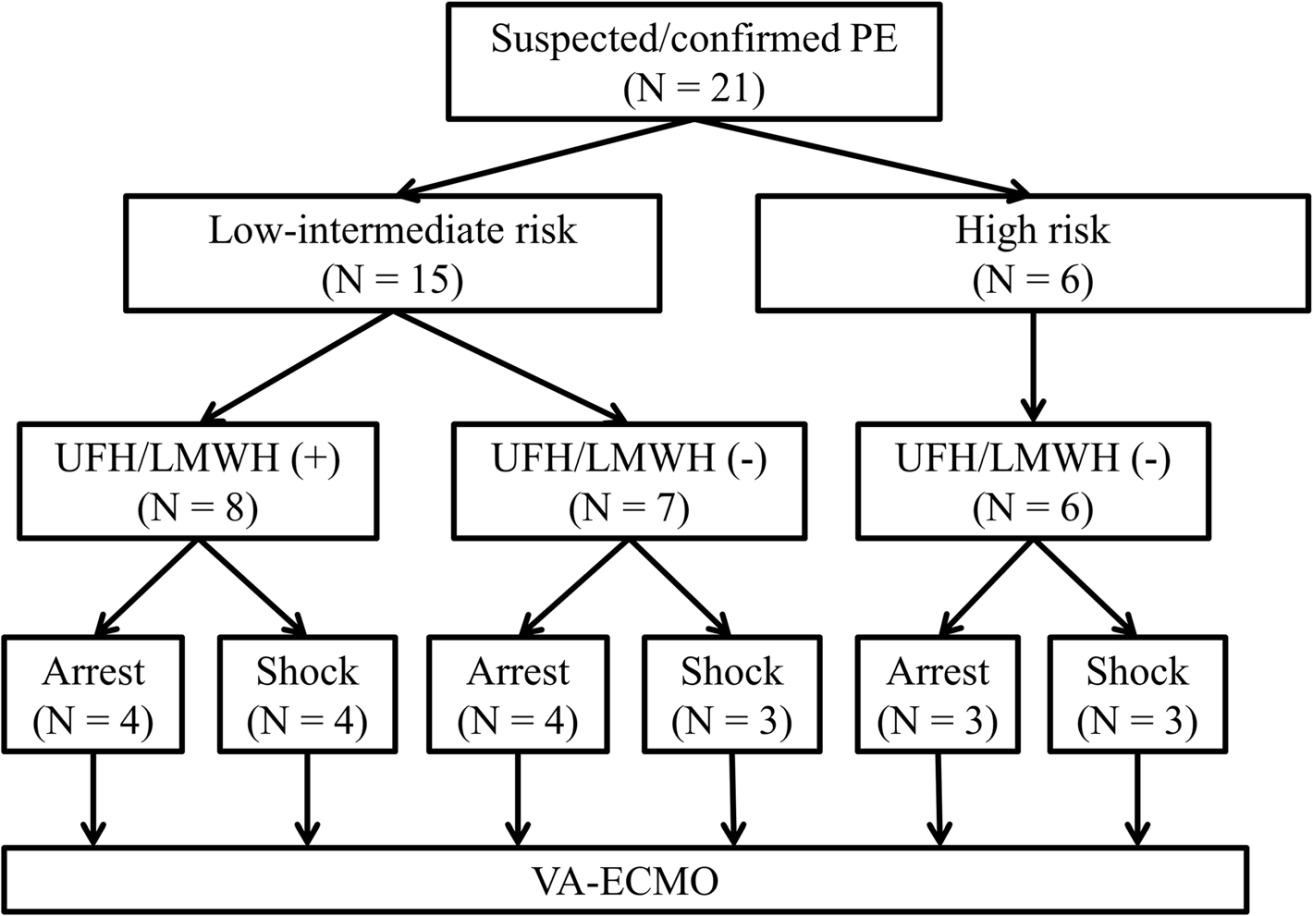
Study flow chart before ECMO support. LMWH, low-molecular-weight heparin; PE, pulmonary embolism; UFH, unfractionated heparin; ECMO, veno-arterial extracorporeal membrane oxygenation

The pre-ECMO clinical condition, risk stratification, and various parameters were summarized in Fig. 1 and Table 2. Before initiation of ECMO, mechanical ventilation was used in four patients for postoperative respiratory assistance and 15 patients for acute respiratory failure. The remaining two patients received emergent intubation during extracorporeal CPR period. Unfractionated heparin or low molecular weight heparin was used in seven patients with confirmed PE and one patient with highly suspected PE before ECMO initiation. More than 90% of patients had ECG findings of RV strain prior to initiating ECMO and imaging signs of RV dysfunction before or during ECMO support. Pre-ECMO blood gases showed severe metabolic acidosis (pH < 7.2) in nine patients (9/18, 50.0%), and moderate to severe hypoxemia (PaO_2_/FiO_2_ < 200 mmHg) in 16 patients (16/18, 88.9%). Elevated cardiac troponin I (≥ 0.04 ng/mL) was seen in 14 patients (14/21, 66.7%). Including six patients presenting cardiogenic shock or cardiac arrest as initial presentation, 17 patients (81.0%) progressed to refractory circulatory collapse within the first day after onset of symptoms, and 16 patients (76.2%) experienced cardiac arrest requiring CPR or refractory cardiogenic shock requiring ECMO support within 2 h after the presentation of refractory circulatory collapse.

**Table 2:** Characteristics of patients at the time of ECMO placement (*N* = 21)

Characteristic	Value
Pre-ECMO mechanical ventilation, n (%)	19 (90.5)
Pre-ECMO UFH/LMWH use	8 (38.1)
Inotrope use, n (%) (before CPR or ECMO initiation)	10 (47.6)
Pre-ECMO condition	
Shock, n (%)	10 (47.6)
Prior cardiac arrest required CPR, n (%)	4 (19.0)
Cardiac arrest during CPR, n (%)	7 (33.3)
Pre-ECMO ECG findings (*n* = 15, within 24 h before initiation of ECMO)	
RV strain, n (%)	11 (73.3)
Sinus tachycardia/atrial fibrillation, n (%)	13 (86.7)
Pre- or post-ECMO finding of RV overload	
Echocardiography (*n* = 20), n (%)	19 (95.0) (pre-ECMO: 13, post-ECMO: 6)
RV/LV diameter ratio on CT (*n* = 19)	1.6 ± 0.6 (range, 0.7 to 2.8)
RV/LV diameter ratio ≥ 0.9, n (%)	18 (94.7) (pre-ECMO: 13, post-ECMO: 5)
Pre-ECMO troponin I level (ng/mL) (within 24 h before ECMO initiation)	0.26 ± 0.39 (range, 0.01 to 1.34)
Cardiac troponin I ≥ 0.04 ng/mL, n (%)	14 (66.7)
Pre-ECMO arterial blood gases (*n* = 18, within 24 h before ECMO initiation)	
pH	7.20 ± 0.18 (range, 6.82 to 7.50)
Bicarbonates (mmol/L)	18.5 ± 7.2 (range, 9.6 to 31.6)
SBE	−9.7 ± 8.6 (range, −23.7 to 4.4)
PaO_2_/FiO_2_ ratio (mmHg)	118.7 ± 93.8 (range, 21.8 to 376.8)
Time from symptoms to shock, hours	25.9 ± 54.3 (range, 0.0 to 238.2)
Time from shock to ECMO, hours	3.4 ± 4.3 (range, 0.4 to 18.3)

*CPR* cardiopulmonary resuscitation, *CT* computed tomography, *ECG* electrocardiogram, *ECMO* veno-arterial extracorporeal membrane oxygenation, *LMWH* low-molecular-weight heparin, *RV/LV* right-to-left ventricular, *SBE* standard base excess, *UFH* unfractionated heparin

### Management and outcomes

**Fig. 2:**
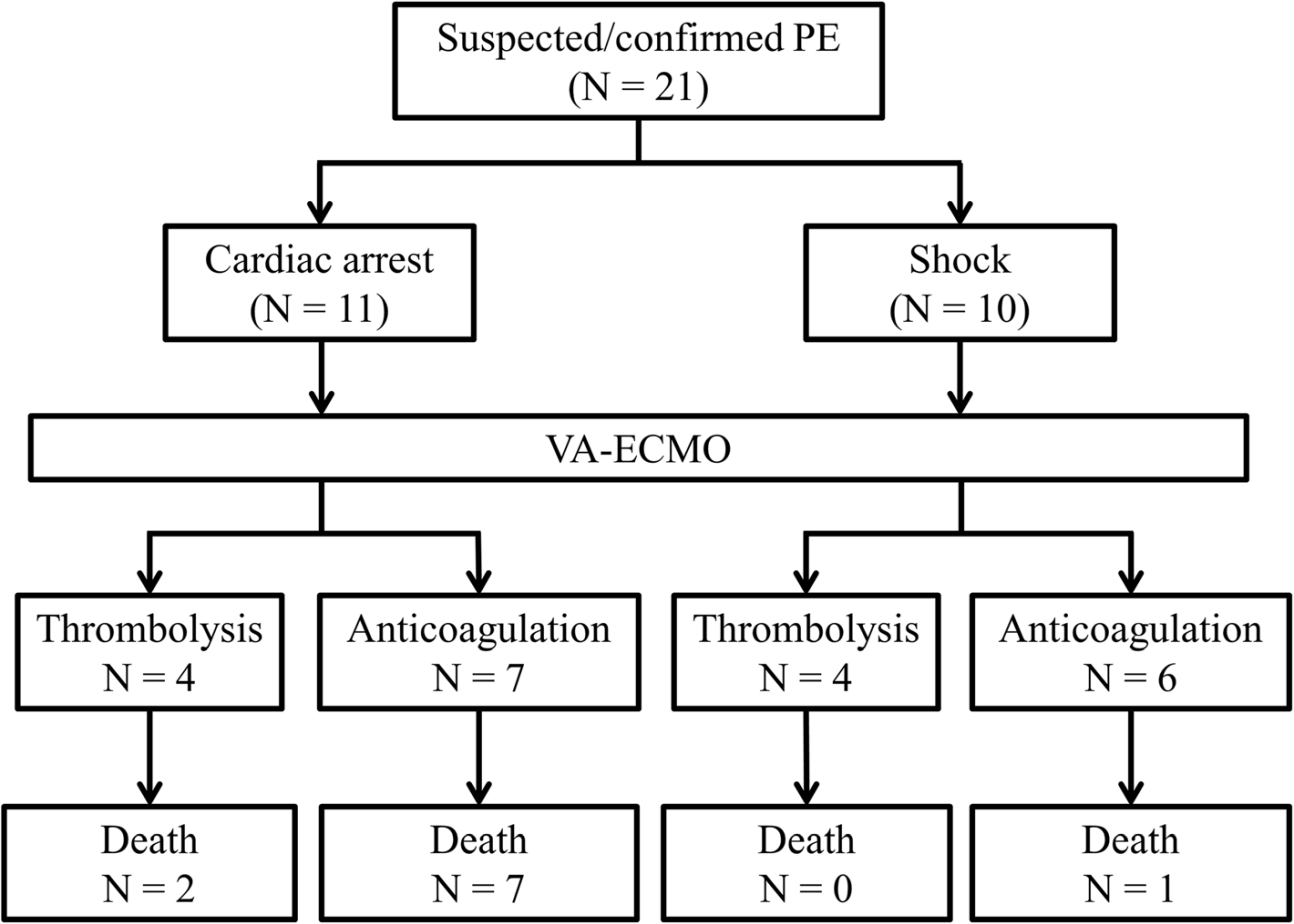
Flow chart describing therapeutic strategy and survival after ECMO support. PE, pulmonary embolism; ECMO, veno-arterial extracorporeal membrane oxygenation

The management strategies and outcomes were detailed in Fig. 2 and Table 3. During ECMO support, eight patients (38.1%) were treated with thrombolytic agents and 13 patients (61.9%) received heparin alone. No patient underwent surgical embolectomy. Among patients receiving thrombolysis, tissue plasminogen activator was administered intravenously by bolus in three patients, catheter-directed thrombolysis with urokinase through central venous catheter was performed in four patients, and ultrasound-enhanced catheter-directed thrombolysis with urokinase through EkoSonic Endovascular System (EKOS Corporation, Bothwell, WA, USA) was performed in one patient.

**Table 3:** Clinical variables and outcomes of patients according to in-hospital survival status

	Survivors (*n* = 11)	Non-survivors (*n* = 10)	p
Age, years	55.4 ± 17.7	54.1 ± 15.1	0.863
Male sex	8 (72.7%)	7 (70.0%)	0.890
Smoking	3 (27.3%)	3 (30.0%)	0.890
BMI (kg/m^2^)	25.8 ± 3.4	24.7 ± 3.9	0.500
Cardiac arrest	2 (18.2%)	9 (90.0%)	0.001
CPR duration, minutes	6.1 ± 14.5	36.3 ± 19.2	
Post-ECMO thrombolytic therapy	6 (54.5%)	2 (20.0%)	0.104
Major complications			
Neurologic impairment	0 (0.0%)	10 (100.0%)	< 0.001
Acute kidney injury required dialysis	0 (0.0%)	7 (70.0%)	0.001
Major bleeding	1 (9.1%)	0 (0.0%)	0.329
ECMO-related complications	1 (9.1%)	1 (10.0%)	0.943
ECMO duration, days	5.0 ± 2.6	5.3 ± 5.8	0.454
MV duration, days	10.3 ± 8.7	6.3 ± 6.6	0.125
ICU LOS, days	15.6 ± 8.3	6.3 ± 6.6	0.012
Hospital LOS, days	29.9 ± 16.0	6.3 ± 6.6	< 0.001

*BMI* body mass index, *CPR* cardiopulmonary resuscitation, *ECMO* veno-arterial extracorporeal membrane oxygenation, *ICU* intensive care unit, *LOS* length of stay, *MV* mechanical ventilation

The in-hospital mortality rate was 47.6%, and the most (90%) leading cause of death was prolonged CPR-related brain death. Compared with non-arrested patients, cardiac arrest prior to ECMO cannulation was a significant predictor of in-hospital mortality (81.8% versus 10.0%, *p* = 0.001). Among survivors, all patients discharged without additional complications, included RV dysfunction.

### Comparison of CT images and cardiac biomarkers for predicting clinical course

**Fig. 3:**
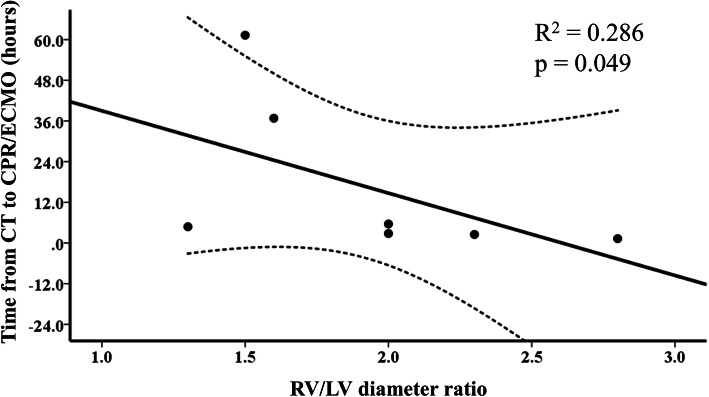
Relationships between the CT values and the time interval from CT exam to CPR or ECMO placement. CPR, cardiopulmonary resuscitation; CT, computed tomography; ECMO, veno-arterial extracorporeal membrane oxygenation; RV/LV, right-to-left ventricular

Thirteen patients who had confirmed diagnosis of PE by CT image before cardiac arrest or ECMO initiation were included for subgroup analysis. The risk classification and various parameters of the patients were detailed in Table 4. All cardiac troponin I testing was performed within 8 h before shock or at the time of shock event. Elevated cardiac troponin I (≥ 0.04 ng/mL) was seen in only 9 patients (69.2%). There was no association between pre-ECMO cardiac troponin-I levels and in-hospital mortality (cut-off point 0.02, area under the curve 0.556; sensitivity 100.00%; specificity 44.44%, *p* = 0.740). The mean time interval from onset of shock to cardiac arrest or ECMO initiation was 2.7 h (range, 0 to 18.3 h). In submassive PE patients, the mean time interval from CT exam to cardiac arrest or ECMO initiation was 3.1 h in patients (*n* = 4) with severe RV dilation (RV/LV diameter ratio ≥ 2) and 34.3 h in patients (*n* = 3) with moderate RV dilation (RV/LV diameter ratio between 1 and 2). In patients with submassive PE, the RV/LV diameter ratio was significantly negatively correlated with the time interval from CT exam to cardiac arrest or ECMO initiation (*p* = 0.049) (Fig. 3).

**Table 4:** Clinical Summary of CT proven PE Patients before CPR or ECMO initiation (*N* = 13)

Case No.	Age, years/Sex	Risk classification	Troponin-I, ng/mL	RV/LV diameter ratio on CT	Time from CT to CPR or ECMO, hours	Time from shock to CPR or ECMO, hours	ECMO indication	Outcome
1	66/F	Low	< 0.02	0.8	149.8	0.0	During CPR	Alive
2	30/M	Submassive	0.05	1.5	61.3	0.2	During CPR	Dead
3	42/M	Submassive	0.05	1.6	36.8	0.6	During CPR	Dead
4	46/F	Submassive	0.181	1.3	4.8	0.0	Previous CPR	Dead
5	60/M	Submassive	0.66	2.3	2.5	0.0	During CPR	Alive
6	42/M	Submassive	0.172	2	5.6	1.9	Shock	Dead
7	45/M	Submassive	0.08	2	2.8	1.0	Shock	Alive
8	72/M	Submassive	< 0.01	2.8	1.3	1.0	Shock	Alive
9	73/M	Massive	< 0.02	0.9	15.2	18.3	Shock	Alive
10	26/F	Massive	1.342	1	2.4	3.3	Shock	Alive
11	56/M	Massive	0.08	1.5	1.4	4.0	Shock	Alive
12	32/F	Massive	1.244	2	1.2	2.8	Shock	Alive
13	83/M	Massive	< 0.02	2.2	1.0	1.4	Shock	Alive

*CPR* cardiopulmonary resuscitation, *CT* computed tomography, *ECMO* veno-arterial extracorporeal membrane oxygenation, *F* female, *LMWH* low-molecular-weight heparin, *M* male, *PE* pulmonary embolism, *RV/LV* right-to-left ventricular, *UFH* unfractionated heparin

## Discussion

Several main findings of this study were as follows: (1) ECMO appears to be an effective circulatory support to salvage patients with life-threatening PE, and excellent outcome can be achieved in patients without cardiac arrest; (2) The combination of ECG, echocardiography, and CT image is useful to determine PE severity. Close monitoring in intensive care unit is recommended in patients with RV dysfunction; (3) In hemodynamically stable patients, the RV/LV diameter ratio measured on CT image is not only a parameter to assess the severity of RV dysfunction, but also a valuable predictor of clinical course.

Despite advances in medical knowledge, PE diagnosis and management remains a challenging clinical problem with a high mortality rate. The interval between onset of symptoms and circulatory collapse may range from minutes to days. 70% of patients with massive PE die within 1 h of developing symptoms [15]. In our study, 76% of patients with hemodynamic instability required CPR or ECMO support within 2 h after the onset of shock. Based on current guidelines [3, 4], primary reperfusion therapy is the first-line therapy for PE patients with cardiogenic shock and ECMO is reserved in patients with refractory cardiogenic shock or cardiac arrest [16]. However, to achieve optimal reperfusion with thrombolysis or embolectomy is difficult technically in real clinical practice. ECMO remains a controversial therapy for massive PE because of unsatisfied results. In a systemic review of 78 patients conducted by Yusuff and colleagues [17], ECMO for selected patients with massive PE is associated with good outcomes. Patients experiencing cardiac arrest had a poor outcome. Two large recent series [8, 16] reported that the survival rate of ECMO for massive PE is 76 to 100% for patients with refractory cardiogenic shock and 13 to 27% for those that required CPR prior to ECMO placement, which is similar to our result (90% versus 18%). There is no doubt that patients presenting in cardiac arrest are associated with worse outcomes. Based on the above findings, massive PE can deteriorate rapidly with progression to cardiac arrest before reperfusion therapy can be achieved. In our observational research during the study period, among 71 patients with massive PE, the use of fibrinolysis before cardiac arrest or ECMO placement was allowed in only four patients (this result was not present in this study). Thus, early, aggressive use of ECMO in patients with massive PE is a reasonable initial management to prevent sudden cardiac arrest [7].

The mechanism of cardiac arrest caused by PE is secondary to RV failure and it thought to occur because of a sharp increase in RV afterload from both mechanical pulmonary arterial obstruction and pulmonary vasoconstriction mediated by neurohumoral factors [12]. Patients without hemodynamic instability but with either sign of RV dysfunction or elevated cardiac biomarker, are classified into the submassive or intermediate risk category [3, 4]. These patients have a higher risk of acute hemodynamic deterioration that close monitoring is recommended [18]. Various objective parameters of RV dysfunction evaluated by ECG, echocardiography, CT or cardiac biomarker have been proposed as predictors of outcome in acute PE [9–14]. In our study of 21 patients, cardiac troponin I was measured at the time of cardiogenic shock or cardiac arrest in 15 patients and within 6 h before shock in four patients. Among those patients, only 13 patients (68.4%) had elevated cardiac troponin I, and five patients (26.3%) had normal cardiac troponin level even in the setting of cardiogenic shock. This means that cardiac troponin I might be insufficient to predictor adverse outcome in real-world clinical practice. Unlike cardiac biomarker, CT image has been used as a standard tool for diagnosis of PE and assessment of severity of RV dysfunction [14]. Many studies reported that RV enlargement was an independent predictor of an adverse outcome, independent of clinical risk factors [14, 19]. However, these reports were only to investigate the association between CT value and adverse outcome. They did not investigate the correlation between CT value and clinical course. In our study, the time interval from diagnosis to cardiac arrest or refractory cardiogenic shock was shorter in patients with severe RV dysfunction, and significant correlation between the RV/LV diameter ratio and the time interval from diagnosis to cardiac arrest or refractory cardiogenic shock requiring ECMO support was also found.

### Limitations

There are several limitations to our study. First, this observational study was a retrospective series without control group for comparison. There was no standard diagnostic strategy and medical treatment protocol for suspected PE after onset of symptoms. Individual treatment and ECMO placement were based on clinical physician experience. Moreover, the relative small sample size may not represent all massive PE patients, and we cannot exclude type 2 error in using of CT values in predicting the clinical course. Although these data suggest that aggressive use of ECMO for patients presenting hemodynamic instability may result in improved outcomes compared with use of ECMO as salvage therapy, the ideal timing of ECMO initiation for these patients may not conclude in this study.

## Conclusions

In real-world practice, patients with submassive or massive PE may rapidly progress to cardiac arrest before reperfusion therapy can be achieved. ECMO appears to be an effective circulatory support to restore hemodynamic status as a bridge to definitive reperfusion therapy or recovery. Early experienced ECMO team approach may reduce ECMO-related complications, resulting in increasing survival rates. However, further randomized, well-controlled, and larger prospective studies are needed to elucidate the role of ECMO in acute PE.

## Data Availability

Not applicable.

## Abbreviations

CPR: Cardiopulmonary resuscitation
CT: computed tomography
ECG: electrocardiogram
ECMO: Veno-arterial extracorporeal membrane oxygenation
PE: Pulmonary embolism
RV: right ventricular
RV/LV: right-to-left ventricular

## References

[1] Donkers-van Rossum AB (2001). Diagnostic strategies for suspected pulmonary embolism. Eur Respir J.

[2] Wood KE (2002). Major pulmonary embolism: review of a pathophysiologic approach to the golden hour of hemodynamically significant pulmonary embolism. Chest..

[3] Jaff MR, McMurtry MS, Archer SL, Cushman M, Goldenberg N, Goldhaber SZ (2011). Management of massive and submassive pulmonary embolism, iliofemoral deep vein thrombosis, and chronic thromboembolic pulmonary hypertension: a scientific statement from the American Heart Association. Circulation..

[4] Konstantinides SV, Torbicki A, Agnelli G, Danchin N, Fitzmaurice D, Galie N (2014). 2014 ESC guidelines on the diagnosis and management of acute pulmonary embolism. Eur Heart J.

[5] Kasper W, Konstantinides S, Geibel A, Olschewski M, Heinrich F, Grosser KD (1997). Management strategies and determinants of outcome in acute major pulmonary embolism: results of a multicenter registry. J Am Coll Cardiol.

[6] Corsi F, Lebreton G, Brechot N, Hekimian G, Nieszkowska A, Trouillet JL (2017). Life-threatening massive pulmonary embolism rescued by venoarterial-extracorporeal membrane oxygenation. Crit Care.

[7] Pasrija C, Kronfli A, George P, Raithel M, Boulos F, Herr DL (2018). Utilization of Veno-arterial extracorporeal membrane oxygenation for massive pulmonary embolism. Ann Thorac Surg.

[8] George B, Parazino M, Omar HR, Davis G, Guglin M, Gurley J (2018). A retrospective comparison of survivors and non-survivors of massive pulmonary embolism receiving veno-arterial extracorporeal membrane oxygenation support. Resuscitation..

[9] Becattini C, Vedovati MC, Agnelli G (2007). Prognostic value of troponins in acute pulmonary embolism: a meta-analysis. Circulation..

[10] Kucher N, Walpoth N, Wustmann K, Noveanu M, Gertsch M (2003). QR in V1--an ECG sign associated with right ventricular strain and adverse clinical outcome in pulmonary embolism. Eur Heart J.

[11] Shopp JD, Stewart LK, Emmett TW, Kline JA (2015). Findings from 12-lead electrocardiography that predict circulatory shock from pulmonary embolism: systematic review and meta-analysis. Acad Emerg Med.

[12] Coutance G, Cauderlier E, Ehtisham J, Hamon M, Hamon M (2011). The prognostic value of markers of right ventricular dysfunction in pulmonary embolism: a meta-analysis. Crit Care.

[13] Lobo JL, Holley A, Tapson V, Moores L, Oribe M, Barron M (2014). Prognostic significance of tricuspid annular displacement in normotensive patients with acute symptomatic pulmonary embolism. J Thromb Haemost.

[14] Becattini C, Agnelli G, Vedovati MC, Pruszczyk P, Casazza F, Grifoni S (2011). Multidetector computed tomography for acute pulmonary embolism: diagnosis and risk stratification in a single test. Eur Heart J.

[15] Stulz P, Schlapfer R, Feer R, Habicht J, Gradel E (1994). Decision making in the surgical treatment of massive pulmonary embolism. Eur J Cardiothorac Surg.

[16] Meneveau N, Guillon B, Planquette B, Piton G, Kimmoun A, Gaide-Chevronnay L (2018). Outcomes after extracorporeal membrane oxygenation for the treatment of high-risk pulmonary embolism: a multicentre series of 52 cases. Eur Heart J.

[17] Yusuff HO, Zochios V, Vuylsteke A (2015). Extracorporeal membrane oxygenation in acute massive pulmonary embolism: a systematic review. Perfusion..

[18] Meyer G, Vicaut E, Danays T, Agnelli G, Becattini C, Beyer-Westendorf J (2014). Fibrinolysis for patients with intermediate-risk pulmonary embolism. N Engl J Med.

[19] Meinel FG, Nance JW, Schoepf UJ, Hoffmann VS, Thierfelder KM, Costello P (2015). Predictive Value of Computed Tomography in Acute Pulmonary Embolism: Systematic Review and Meta-analysis. Am J Med.

